# Editorial: Early social experience: impact on early and later social-cognitive development

**DOI:** 10.3389/fpsyg.2023.1268725

**Published:** 2023-08-14

**Authors:** Sarah A. Gerson, Caroline Junge, Marlene Meyer

**Affiliations:** ^1^Cardiff University Centre for Human Developmental Science, School of Psychology, Cardiff University, Cardiff, United Kingdom; ^2^Department of Experimental Psychology, Helmholtz Institute, Utrecht University, Utrecht, Netherlands; ^3^Donders Institute for Brain, Cognition and Behaviour, Radboud University, Nijmegen, Netherlands

**Keywords:** early experience, social cognition, early childhood, language development, longitudinal research, developmental cascades

Humans are social beings, and engage in social interactions from early in life. Yet, grasping early social-cognitive development is incredibly complex: besides a child's genetic predisposition, there are a wide variety of environmental experiences, across nested time scales, which are shaping a child's social-cognitive development (e.g., Masten and Cicchetti, 2010; d'Souza et al., 2017; Junge et al., 2020; Tamis-LeMonda, 2023). To further capture the broad range of social experiences, this Research Topic aimed to bring together research that addressed how infants' and young children's social environments shape their early and later social and cognitive development from a variety of perspectives, including both empirical and theoretical papers, spanning typical and atypical populations.

This Research Topic showcases the complexity of social-cognitive development in a variety of ways. For example, it spans a wide range of social experiences: it includes empirical papers ranging from micro-level factors such as parenting styles (He et al.; Iwasaki et al.; Kim; Krijnen et al.; Ramos et al.) and other daily life experiences (Guellai et al.; McCall et al.) to macro-level experiences such as COVID-19 (Almeida et al.; Wermelinger et al.). In addition, it encompasses empirical research on a variety of neurotypical and neurodivergent populations (ASD: He et al.; Adoptees: Ramos et al.; Preterm infants: Krijnen et al.). There is further a great variety in empirical methods employed to capture this development: spanning behavioral observations, eye-tracking (He et al.), and network analyses (Burke et al.). Finally, this Research Topic covers not only empirical research but also provides a case report (McCall et al.), a theoretical paper (Belteki et al.), and a review (Guellai et al.). All in all, this Research Topic fully embraces the complexity of social experiences, as it considers a variety of experiences, outcomes, populations, methods, and approaches, all of which contribute in shaping early development.

As we framed this Research Topic in terms of outcomes on social-cognitive development, authors defined this in various, compelling manners. They examined the influence of early experience upon self-esteem (Kim), emotion labeling (Wermelinger et al.), language development (Bazhydai et al.; Belteki et al.; He et al.), curiosity (Iwasaki et al.), social competence and psychosocial behavior (Krijnen et al.; Ramos et al.; Zhu et al.), and attention and cognitive development more broadly defined (Almeida et al.).

In addition to the scientific impact, these outcomes are also relevant because of the potential interest beyond academia in terms of implications for society. For example, the interest in children's screentime and media exposure (Almeida et al.; Guellai et al.) is a pressing issue for caregivers, policy-makers, and educators alike. Moreover, the consequences of various parenting styles and the development of social networks (Burke et al.; He et al.; Iwasaki et al.; Kim; Krijnen et al.; Ramos et al.) may help inform caregivers and educators. The potential impact of foster care, adoption, and government policies relating to children in care (McCall et al.) is relevant for practitioners and policy-makers. The effects of COVID-19 (Almeida et al.; Wermelinger et al.) are important to consider both for facilitating recovery and future-proofing against potential issues for children and families when global crises may arise.

The impact for both academia and society is even stronger given the striking diversity of researchers who contributed to this Research Topic. The articles come from authors across five continents (Africa, Asia, Europe, North America, and South America) and 14 countries. Including a diversity of both researchers and participants is critical for moving global developmental science forward (Apicella et al., 2020; Moriguchi, 2022; Singh et al., 2023). As such, we hope that this Research Topic models the move toward embracing not only a complexity of methodologies but also the benefits of taking an international view of development and including both researchers and children and families from diverse regions across the globe.

## Author contributions

SG: Conceptualization, Writing—original draft, Writing—review and editing. MM: Conceptualization, Writing—original draft, Writing—review and editing. CJ: Conceptualization, Writing—original draft, Writing—review and editing.
